# *Clostridioides difficile* in Food-Producing Animals in Romania: First Study on the Prevalence and Antimicrobial Resistance

**DOI:** 10.3390/antibiotics11091194

**Published:** 2022-09-03

**Authors:** Corina Beres, Liora Colobatiu, Alexandra Tabaran, Romolica Mihaiu, Cristian Iuhas, Marian Mihaiu

**Affiliations:** 1 Department of Animal Breeding and Food Science, Faculty of Veterinary Medicine, University of Agricultural Sciences and Veterinary Medicine, Manastur Street No. 3/5, 400372 Cluj-Napoca, Romania; 2 Department of Medical Devices, Faculty of Pharmacy, Iuliu Hatieganu University of Medicine and Pharmacy, Victor Babes Street No. 8, 400012 Cluj-Napoca, Romania; 3 Department of Management, Faculty of Economic Sciences and Business Administration, Babes Bolyai University, Mihail Kogalniceanu Street No.1, 400084 Cluj-Napoca, Romania; 4 Department of Obstetrics and Gynecology, Faculty of Medicine, Iuliu Hatieganu University of Medicine and Pharmacy, Victor Babes Street No. 8, 400012 Cluj-Napoca, Romania

**Keywords:** *Clostridioides difficile*, *Clostridioides difficile* infection, toxins, antimicrobial resistance, food-producing animals, biosecurity, zoonosis

## Abstract

At present, the epidemiology of the gastrointestinal disease caused by *Clostridioides difficile* (*C. difficile*) is starting to be slowly elucidated internationally, although information about the bacteria in the food supply chain is insufficient and, in many countries, even absent. The study was conducted in order to investigate the prevalence of *C. difficile* isolated from animal feces, as well as to determine the antimicrobial susceptibility of such isolates. The presence of antibiotic resistance determinants has also been evaluated. Overall, a total of 24 (12.5%) *C. difficile* isolates were recovered (out of the 192 samples collected), the highest percentage of positive isolates being detected in the fecal samples collected from piglets (25%). The majority of the isolates recovered in the current study proved to be toxigenic. Moreover, all *C. difficile* isolates were susceptible to vancomycin, although a large proportion of the porcine isolates (50%) were resistant to levofloxacin. The *tetW* and *erm(B)* genes have also been identified in the porcine isolates. In conclusion, this is the first analysis of the prevalence of *C. difficile* in food-producing animals in Romania, and it adds further evidence about the possible role of animals as a source of resistant *C. difficile* strains and a reservoir of antimicrobial resistance determinants.

## 1. Introduction

*Clostridioides difficile* (*C. difficile*) is an anaerobic, Gram-positive, spore-forming, enteric pathogen that causes gastrointestinal infections in humans (*C. difficile* infection-CDI) [1,2]. CDI is a toxin-mediated disease of the colon, which usually manifests as a wide spectrum of conditions, from self-limiting diarrhea to life-threatening colitis [2]. *C. difficile* can express up to three toxins: toxin A (TcdA), toxin B (TcdB), as well as the *C. difficile* transferase (CDT) binary toxin [3,4]. To date, *C. difficile* has been isolated from different sources, such as food animals (pigs, cattle, sheep, poultry), retail meat (veal, beef, pork, lamb, chicken, and turkey), as well as seafood, vegetables, and the environment (both household and natural) [1,2,5,6,7,8]. Initially, it was considered that infection with *C. difficile* was primarily hospital-acquired, it being most frequently associated with the exposure to broad-spectrum antimicrobials that generally disrupt the microbiota of the gastrointestinal tract [5]. At present, the epidemiology of the gastrointestinal disease caused by *C. difficile* is starting to be slowly elucidated internationally. Taking into consideration the emergence of community-associated cases of infection, as well as whole-genome sequencing data suggesting many of the hospital CDI cases are significantly different from one another, it has been considered that distinct sources of *C. difficile* outside the hospital, such as food animals, retail food, and the environment, may represent an important reservoir of toxigenic *C. difficile* and might be playing a major and previously unrecognized role in the transmission of CDI [5].

Food animals are recognized carriers of *C. difficile* [9]. Pigs are the farm animals that have been most commonly studied in Europe with regard to CDI [10]. It has been particularly noticed and reported that neonatal animals, such as piglets or calves, are more frequently intestinally colonized with *C. difficile* at slaughterhouses compared to fully-grown animals [11]. The global prevalence of *C. difficile* in piglets is generally considered to be high, ranging from 8.4% in the United States of America to 67.2% in Austria, 73.15% in Germany, 78.35% in Belgium, and 85.1% in Taiwan [5,12,13,14]. Moreover, toxin detection ranging from 1.4% to 96% has been reported in piglets in several previous studies [11]. The most prevalent ribotypes identified in piglets are 013, 014, 015, 078, and 126 [11].

Different ribotypes such as 027, 053, 017, and 078 have been described in human isolates in Europe and also in farm animals and meat (especially ribotypes 078 and 017, both being able to cause severe human intestinal diseases). New ribotypes are continuously being detected [15,16]. Recently, *C. difficile* has been defined as a new zoonotic agent, even if, according to some authors, objective evidence for foodborne transmission is still absent. *C. difficile* ribotype 078 has emerged at the same time in humans and livestock, and zoonotic transmission seems probable, as genotypes and diseases resemble each other [17]. Moreover, studies have demonstrated similarities between *C. difficile* isolates from animals or food and clinical isolates, thus suggesting zoonotic transmission [12,18,19,20,21]. However, the zoonotic aspect is not yet completely clarified, and further analysis is needed to reveal the exact transmission routes.

Information about *C. difficile* in the food supply chain is insufficient and, in many countries, even absent. The bacteria are not yet integrated into the few existing integrated surveillance systems, and they are rarely tested for antimicrobial susceptibility; therefore, little is known regarding antimicrobial-resistant *C. difficile*, especially in animals and foods of animal origin. There are almost no data available about the prevalence, circulation, and antimicrobial susceptibility of *C. difficile* strains of food or animal origin in Romania.

Nevertheless, there is now compelling evidence demonstrating the relevance of *C. difficile* to the One Health concept. Three independent problems requiring an integrative solution are currently being described: a human health issue, an animal health issue, and an environmental issue [5].

The study was conducted in order to investigate the prevalence of *C. difficile* isolated from animal feces, as well as to determine the antimicrobial susceptibility of such isolates. The presence of antimicrobial resistance determinants has also been evaluated.

## 2. Results

### 2.1. Prevalence of C. difficile

A total of 24 (12.5%) *C. difficile* isolates were recovered from the 192 analyzed samples. Overall, the highest percentage of positive isolates was detected in the fecal samples collected from piglets (25%). A low percentage of *C. difficile* isolates was also recovered from the beef cattle and veal calves’ fecal samples (4.16% and 4.41%, respectively) (Table 1).

### 2.2. Toxin Genes Profiling

The results regarding the virulence gene profiles are presented in Table 1. A large proportion of the isolates recovered from piglet feces were toxigenic (95%). The results indicated that 2 (2/20, 10%) of these isolates carried the *tcdA, tcdB* (*tcdA^+^*, *tcdB^+^*), and *cdtA/B* (*cdtA/B^+^*) genes, while 17 isolates (17/20, 85%) were only positive for *tcdA* and *tcdB*. Among the isolates detected in the fecal samples collected from veal calves, one (1/3, 33%) carried the *tcdA* and *tcdB* genes.

### 2.3. Antimicrobial Susceptibility Testing

The susceptibility profiles of the *C. difficile* isolates grouped by animal species are presented in Table 2.

According to the MIC interpretative breakpoints applied in the study, 60% (12/20) of the porcine isolates were resistant to tetracycline, while 50% (10/20) showed resistance to levofloxacin. A small proportion of these also proved to be resistant to erythromycin (4/20, 20%). Among the *C. difficile* isolates recovered from veal calves, one isolate was resistant to both tetracycline and levofloxacin. Vancomycin was active against all isolates of *C. difficile*.

### 2.4. The Presence of Antimicrobial Resistance Determinants

In total, seven *C. difficile* (7/20, 35%) isolates recovered from the porcine fecal samples carried the *tetW* gene. These were also resistant to tetracycline. Moreover, two porcine isolates also showed an *erm(B)* gene. The presence of the *tetM* gene has not been detected in the *C. difficile* isolates included in the study.

## 3. Discussion

The emergence of epidemic strains of *C. difficile* that proved to be resistant to multiple antimicrobial agents has prompted considerable effort in elucidating the epidemiology of these bacteria, most of it being dedicated to identifying potential sources as well as transmission routes for the community-acquired CDI. In this context, farm animals are receiving increasing attention as possible sources of toxigenic *C. difficile* [9].

Overall, a total of 24 (12.5%) *C. difficile* isolates were recovered from the 192 analyzed samples, the highest percentage of positive isolates being detected in the fecal samples collected from piglets (25%). This result is consistent with various studies performed in Europe and North America, which reported a prevalence ranging from 0.5% to 20%, although higher isolation rates have also been identified, particularly in Australia and Korea (60% and 45%, respectively) [2,22,23,24,25,26,27]. The isolation levels of *C. difficile* that have been reported so far might seem quite contrasting; however, it is generally considered and reported that such differences may be due to methodological, geographical, or seasonal variations. The age of the animals also significantly influences the recovery of *C. difficile* [2,28].

To date, *C. difficile* has been isolated from different sources, including food animals or retail meat, as well as seafood, vegetables, and the environment. Moreover, due to recent advances in whole-genome sequencing technologies, studies that compared human and animal *C. difficile* isolates have shown that such strains are genetically closely related and, in some cases, even indistinguishable, thus suggesting possible zoonotic transmission between animals and humans [5,19,29,30].

The majority of the isolates recovered in the current study proved to be toxigenic (10% of the porcine isolates carried the *tcdA, tcdB*, and *cdtA/B* genes, while 85% were positive for *tcdA* and *tcdB*). Among the isolates detected in the fecal samples collected from veal calves, one of them carried the *tcdA* and *tcdB* genes. Therefore, in this study, *tcdA^+^ tcdB^+^ C. difficile* was the predominant profile. In general, most *C. difficile* strains produce both *tcdA* and *tcdB* toxins, while some strains only produce *tcdB* or even no toxins at all. The prevalence of the binary toxin-encoding genes (*cdtA* and *cdtB*) was high but in accordance with previous studies. Even though the role of these genes in the pathogenesis of CDI is not yet clear, the binary toxin is considered to be responsible, at least in part, for community-acquired CDI in humans [3,27,31].

In the context of the frequent use of antimicrobial agents in the treatment of both animals and humans, the main concern remains the emergence of antimicrobial-resistant bacteria, which, unfortunately, has increased among many pathogenic anaerobic bacteria as well [32].

In the current study, the Etest (bioMérieux, Marcy l’Etoile, France) was used in order to determine the susceptibility to tetracycline, erythromycin, clindamycin, levofloxacin, vancomycin, and metronidazole. At the current moment, the methodology for the antimicrobial susceptibility testing of anaerobes has not been standardized, at least not to the same extent as for aerobic microorganisms. In this context, the Etest represents a practical alternative for the determination of the MIC of anaerobic bacteria, providing results that are consistent with the MIC determined using the standard agar diffusion method [32,33].

All *C. difficile* isolates recovered in our study proved to be susceptible to vancomycin, while one isolate from beef cattle feces was resistant to metronidazole. Metronidazole has long been used as a first-line antimicrobial in the treatment of moderate to severe CDI, although it is no longer recommended in the treatment of CDI whenever vancomycin of fidaxomicin is available. According to the recent updated guidelines regarding the management of CDI, fidaxomicin is currently the preferred drug for the treatment of initial infection with *C. difficile* (when available and feasible), with oral vancomycin considered as an acceptable alternative [34,35]. Our results are consistent with the ones reported in other studies, indicating that the occurrence of metronidazole- or vancomycin-resistant strains remains very low.

A large proportion of the porcine isolates (50%) were resistant to levofloxacin, a third-generation fluoroquinolone. Fluoroquinolone resistance is quite common among human and animal isolates of *C. difficile* and might be due to the selective pressures derived from the extensive use of this particular class of antimicrobial agents in hospitals, therefore resulting in the clonal expansion of resistant strains. It has even been suggested that pigs may have acquired fluoroquinolone-resistant strains from humans, but further investigation on this matter is clearly required [36,37]. Nevertheless, similarly, the unreasonable use of antimicrobials in animal husbandry may also contribute to the expansion of drug-resistant strains in farms [36,37]. Resistance to fluoroquinolones in *C. difficile* is determined by alterations in the quinolone resistance determining region (QRDR) of either GyrA or GyrB, the DNA gyrase subunits [38]. In vitro experiments have proved that exposure to levofloxacin might induce a high frequency of selection for GyrA and GyrB drug-resistant mutants in previously susceptible strains [33].

Interestingly, 60% of the isolates recovered from the fecal samples collected from piglets also proved to be resistant to tetracycline, 35% of these also carrying the *tetW* gene. Recent papers indicate that the resistance of *C. difficile* to tetracycline varies among countries, from 2.4% to 41.67%, although it is not so prevalent among *C. difficile* clinical isolates. Although *tetM* seems to be the most widespread class in *C. difficile*, other *tet* genes have also been identified—in particular, the copresence of both *tetM* and *tetW* in isolates of human and animal origin.

Four porcine isolates were found to be resistant to erythromycin, two of them also showing an *erm(B)* gene. Macrolides, as well as fluoroquinolones (especially enrofloxacin), are often used in swine and cattle, while the presence of an *erm(B)* gene may be problematic, as it was reported to play a major role in the resistance to the macrolide-lincosamide-streptogramin B (MLSB) group of antibiotics [26,32].

Almost all of the isolates recovered from the fecal samples collected from piglets (except for one), as well as one veal calf isolate which proved to be resistant to the antimicrobials used in the study, were also toxigenic.

## 4. Materials and Methods

### 4.1. Sampling

A total of 192 samples of animal feces (100 from piglets, 24 from beef cattle, and 68 from veal calves) were collected from January 2021 to March 2022 from three geographically distinct farms located in the center of Romania. The fecal samples (approximately 50 g) were collected aseptically, directly from the rectum, transported to the laboratory under ambient conditions, stored at 4 °C, and processed within 24 h.

### 4.2. C. difficile Isolation

The fecal samples were plated directly onto *C. difficile* ChromID^TM^ (bioMérieux, Marcy l’Etoile, France). This is a chromogenic medium containing taurocholate and a chromogen mix, allowing for the isolation and identification of *C. difficile* strains in 24 h. All plates were then incubated in an anaerobic chamber (Don Whitley Scientific Ltd., Shipley, West Yorkshire, UK) at 37 °C for 24 h in an atmosphere containing 80% nitrogen, 10% hydrogen, and 10% carbon dioxide. After incubation, microbial growth and the presence of typical colonies of *C. difficile* (grey to black, with an irregular or smooth border) were observed.

### 4.3. Toxinotyping of Isolates

The DNA was extracted using the QIAamp^®^ DNA Mini Kit (Qiagen, Hilden, Germany), according to the manufacturer’s protocol. The expression of the genes that encode for toxin A and toxin B (*tcdA* and *tcdB*, respectively), as well as of the two components of the binary toxin (CDT) (*cdtA* and *cdtB*), was detected by Real-Time PCR, as previously reported [39].

### 4.4. Antimicrobial Susceptibility Testing

The susceptibility to tetracycline, erythromycin, clindamycin, levofloxacin, vancomycin, and metronidazole was determined using the Etest (bioMérieux, Marcy l’Etoile, France), according to the protocol indicated by the manufacturer. The MIC interpretative breakpoints defining resistance that were used in the study were defined by the Clinical and Laboratory Standards Institute (CLSI), except for erythromycin (in which case an MIC breakpoint that was previously reported was used). The MIC interpretative breakpoints applied were the following: tetracycline ≥ 16 μg/mL, clindamycin ≥ 8 μg/mL, levofloxacin ≥ 8 μg/mL, vancomycin > 2 μg/mL, metronidazole > 2 μg/mL, and erythromycin > 256 μg/mL [40,41]. *Bacteroides thetaiotaomicron* ATCC 29741 and *C. difficile* ATCC 700057 were used as quality controls, as well as to confirm that the anaerobic conditions were achieved during the incubation process.

### 4.5. Detection of Antibiotic Resistance Determinants

Multiplex PCR was performed in order to amplify the genes *tetM* and *tetW* (coding for ribosomal protection proteins and conferring resistance to tetracycline), as well as the *ermB* genes (conferring resistance to the MLSB group of antibiotics), using the related primers, as previously described [26,42,43].

## 5. Conclusions

In conclusion, this is the first analysis of the prevalence of *C. difficile* in food-producing animals in our country, providing a baseline for the future surveillance of the antimicrobial resistance of *C. difficile* in food-producing animals, food, and the environment in Romania. A further, more complex study including human *C. difficile* isolates should be performed in order to assess a possible role of food animals as source of resistant *C. difficile* strains and a reservoir of antimicrobial resistance determinants.

## Figures and Tables

**Table 1:** Recovery of *Clostridioides difficile*.

Sources	n	Sample Isolation Rates (%)	Toxigenic Isolates (%)		Non-Toxigenic Isolates (%)
			*tcdA^+^, tcdB^+^, cdtA^+^/B^+^*	*tcdA^+^, tcdB^+^*	
Piglets	100	20/100 (25)	2/20 (10)	17/20 (85)	1/20 (5)
Beef cattle	24	1/24 (4.16)	0/24 (0)	0/24 (0)	1/1 (100)
Veal calves	68	3/68 (4.41)	0/3 (0)	1/3 (33)	2/3 (66)

**Table 2:** Susceptibility profiles of the *Clostridioides difficile* isolates grouped by animal species.

	Antimicrobials ^1^					
	TE	EM	CM	LE	VA	MZ
Piglet (n = 20)	12	4	0	10	0	0
Resistance (%)	60	20	0	50	0	0
Beef cattle (n = 1)	0	0	0	1	0	1
Resistance (%)	0	0	0	100	0	100
Veal calves (n = 3)	1	0	0	1	0	0
Resistance (%)	33.33	0	0	33.33	0	0

^1^ TE—Tetracycline, EM—Erythromycin, CM—Clindamycin, LE—levofloxacin, VA—vancomycin, MZ—metronidazole.
